# A dermal backflow atlas of lymphatic dysfunction in breast cancer-related lymphedema with machine learning-based stage prediction

**DOI:** 10.1007/s10549-026-08093-2

**Published:** 2026-10-07

**Authors:** Lalida Sutejitsiri, Hiroo Suami, Poul M F Nielsen, Anthony R J Phillips, Hayley M Reynolds

**Affiliations:** 1 Auckland Bioengineering Institute, The University of Auckland, Auckland, New Zealand; 2 Australian Lymphedema Education Research and Treatment (ALERT) Programme, Department of Health Sciences, Faculty of Medicine, Health and Human Sciences, Macquarie University, Sydney, Australia; 3 Engineering Science and Biomedical Engineering, Faculty of Engineering, The University of Auckland, Auckland, New Zealand; 4 School of Biological Sciences, Faculty of Science, The University of Auckland, Auckland, New Zealand

**Keywords:** Breast cancer-related lymphedema, Indocyanine green lymphography, Machine learning, Dermal backflow, Image analysis

## Abstract

**Purpose:**

Breast cancer-related lymphedema (BCRL) is a chronic condition caused by impaired lymphatic drainage following cancer treatment, manifesting as fluid accumulation, adipogenesis, and lymph reflux to the skin (dermal backflow). The affected regions are diverse and therefore, improved understanding of the spatial distribution of lymphatic dysfunction may facilitate earlier detection and disease monitoring. This study aimed to develop a spatial atlas of dermal backflow in BCRL and apply machine learning (ML) models to predict disease severity.

**Methods:**

Data from 282 female BCRL patients (289 affected limbs) were obtained from the Australian Lymphedema Education, Research and Treatment (ALERT) Centre Databank, including indocyanine green (ICG) lymphography studies and clinical information. A spatial atlas of dermal backflow prevalence in the upper extremities was constructed by co-registering and segmenting clinician-annotated ICG diagrams. Five ML models were developed to classify MD Anderson Cancer Center (MDACC) lymphedema stage, incorporating dermal backflow percentage and clinical features.

**Results:**

The atlas revealed consistent spatial patterns of dermal backflow, with the highest prevalence in the wrist, forearm, and the olecranon region. Subgroup-specific heat maps stratified by MDACC stage, L-Dex ratio, limb volume difference, drainage regions, and hand dermal backflow further demonstrated distinct spatial variations with disease severity. The inclusion of dermal backflow percentage improved ML prediction of MDACC stage, with the best performance achieved by multinomial logistic regression and random forest models.

**Conclusion:**

Dermal backflow prevalence revealed non-uniform, region-specific patterns in BCRL patients, which can be visualized using spatial atlases. Integrating quantitative imaging features into ML models improves BCRL staging and supports objective, data-driven disease assessment.

**Supplementary Information:**

The online version contains supplementary material available at https://doi.org/10.1007/s10549-026-08093-2.

## Introduction

Breast cancer-related lymphedema (BCRL) is a common and clinically debilitating complication, affecting approximately 20% of breast cancer survivors following treatment [1]. The likelihood of developing BCRL is associated with disruption to lymphatic anatomy and function, particularly following lymph node surgery and radiotherapy [2, 3]. Despite well-established risk factors, the onset and progression of BCRL remain highly variable and difficult to predict at the individual level [4], which poses a major challenge for early detection and timely intervention.

Recently, the MD Anderson Cancer Center (MDACC) scale was developed to stage lymphedema severity. This used indocyanine green (ICG) lymphography, incorporating qualitative assessment of dermal backflow patterns and presence of functional lymphatic vessels [5] (see Supplementary Fig. 1). While clinically informative, it is inherently subjective and dependent on clinician interpretation [6], limiting its ability to fully capture the spatial extent, heterogeneity, and severity of lymphatic dysfunction across the limb. At the same time, the growing adoption of ICG lymphography has generated large volumes of image-based data containing complex spatial information that cannot be easily quantified by visual inspection alone [7–10]. Image analysis techniques and machine learning (ML) offer a potential solution by enabling automated quantification of dermal backflow and integration of imaging-derived features with clinical variables to objectively assess lymphedema severity. Such approaches may improve the accuracy and reproducibility of lymphedema staging and provide additional insight into disease severity, cofounding factors, and progression.

At the Australian Lymphedema Education, Research and Treatment (ALERT) Centre, Macquarie University in Sydney, Australia, a large dataset of clinician-annotated ICG lymphography diagrams and associated clinical data has been established [11, 12]. This dataset represents a unique resource for investigating the spatial distribution of dermal backflow at the cohort level and for evaluating the role of quantitative imaging features in predicting disease severity. In this study we aimed to improve understanding of lymphatic dysfunction in BCRL by developing a spatial atlas of dermal backflow distribution using annotated ICG lymphography diagrams from the ALERT Centre, and by evaluating the performance of ML models to predict MDACC stage using clinical variables alongside dermal backflow metrics derived from ICG diagrams.

## Methods

### Participant data

All patient data used in this study were provided by the ALERT Centre, Macquarie University. Data were extracted and exported from the ALERT Databank via REDCap (Research Electronic Data Capture) by interrogating the database for all patients with confirmed BCRL who had undergone an ICG lymphography study and demonstrated dermal backflow as a diagnostic criterion. ICG lymphography data were exported as image files, and clinical data were exported in CSV format. All data were de-identified prior to analysis to ensure patient confidentiality.

**Fig. 1:**
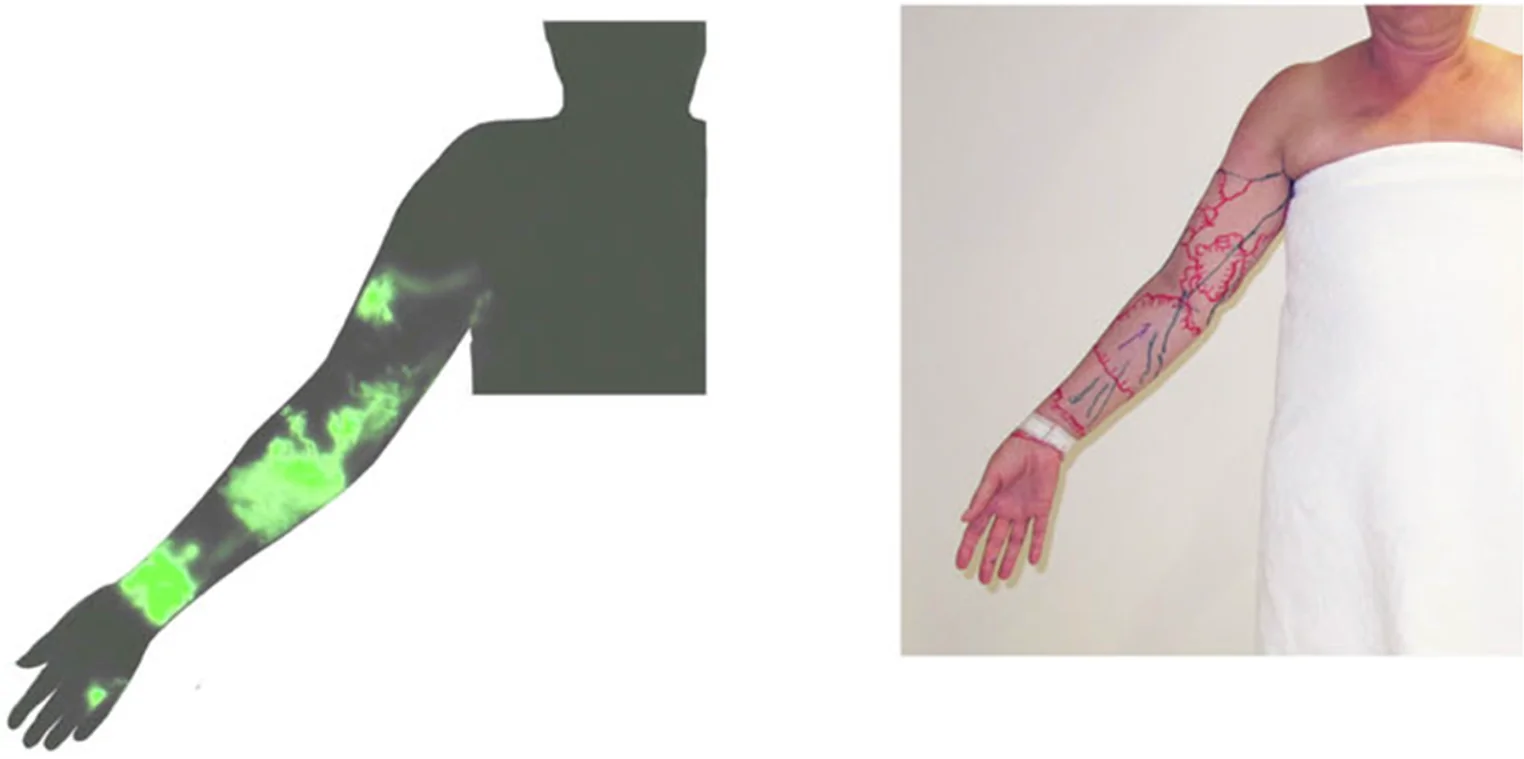
Two types of images obtained from the ALERT ICG lymphography protocol, adapted from [12]. Near-infrared (NIR) fluorescence image showing the ICG signal **(left)** of a patient’s left arm; and visible light image **(right)** of the same patient’s left arm with demarcations: red shows dermal backflow areas, while green shows the patent lymphatic vessels

**Fig. 2:**
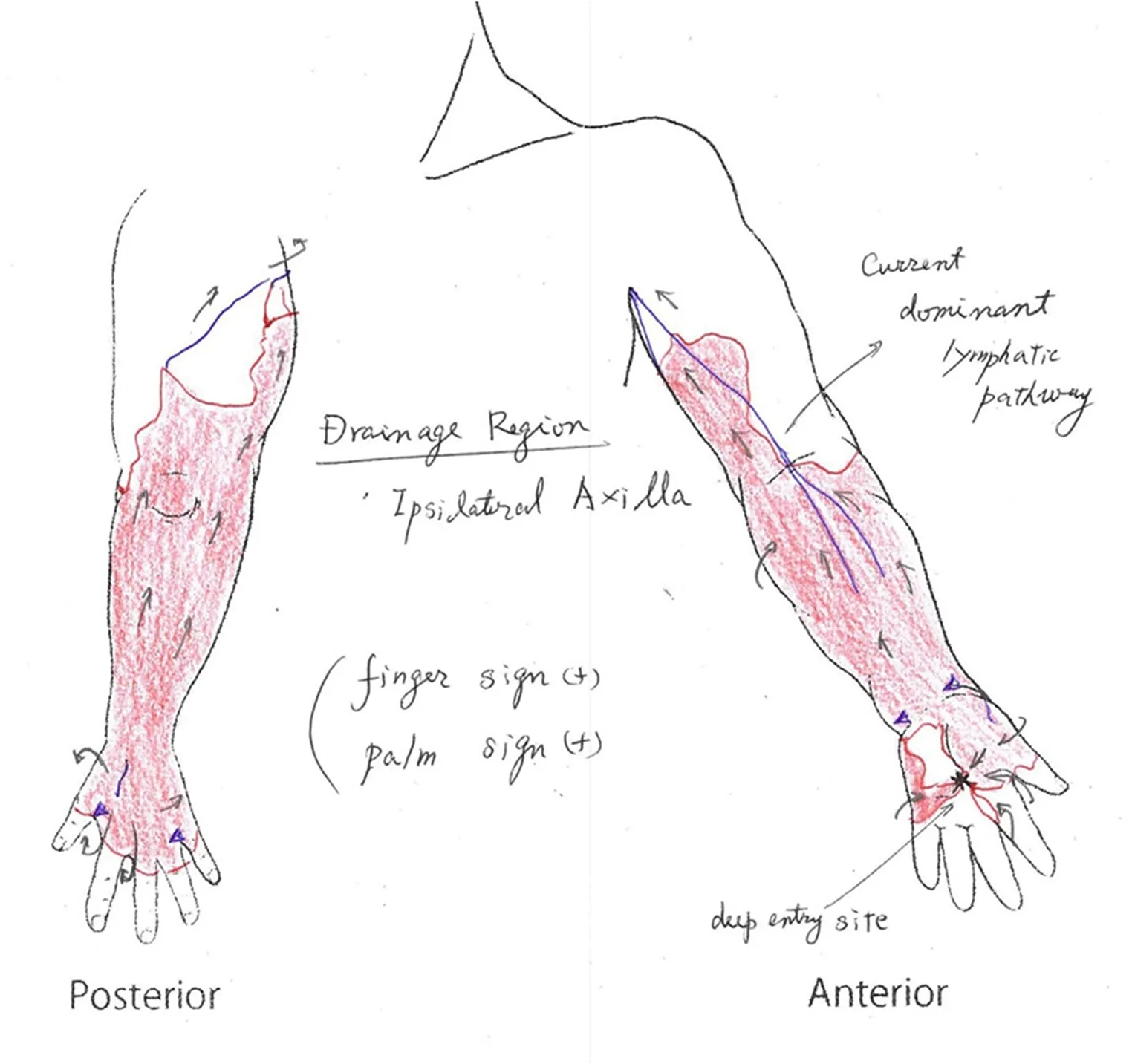
Example clinician-drawn ICG diagram showing posterior and anterior views of a patient’s left arm, where dermal backflow regions are shown in red with annotated lymphatic drainage pathways. This patient was classified as having MDACC stage 3 lymphedema [5]

ICG lymphography data captured two image types, as shown in Fig. 1: near-infrared (NIR) fluorescence images capturing the ICG signal; and visible light images. The ICG data was all acquired by four clinicians at ALERT, who each completed competency training to ensure they followed a uniform protocol. During the imaging studies, each clinician drew lymphatic drainage pathways observed on the patient’s skin directly, including marking regions of dermal backflow which reached stability after approximately one hour of the ICG imaging session. A summary of findings for each patient study was drawn immediately onto a paper template image by the clinician who performed the study, with reference to ICG images and photographic images of the patient. The synthesized summary image was later digitized and stored as a 24-bit RGB JPEG image at 300 DPI (see Fig. 2). MDACC staging for each patient was determined by the same clinician, with reference to the ICG staging scale [5]. These diagrams included dermal backflow regions highlighted in red, the direction of lymphatic drainage pathways was indicated by black arrows, and delineations indicated by blue lines marking the nodal basin(s) to which lymph fluid drains: the ipsilateral axilla, clavicular, parasternal, contralateral axilla, and parascapular regions. The ALERT Centre provided four standard reference templates with anterior and posterior views, covering the left arm, the right arm, the left arm and torso, and the right arm and torso.

Clinical data exported included the following variables: age, year of initial breast cancer diagnosis, duration of lymphedema, MDACC stage, presence of dermal backflow in the hand, lymphatic drainage region(s), breast cancer treatment history, lymphadenectomy status, affected limb(s), limb volume difference (LVD), bioimpedance spectroscopy (L-Dex score), and body mass index (BMI).

### Heat maps of dermal backflow

#### Preprocessing and standardisation of ICG diagrams

To create spatial heat maps of dermal backflow, patient ICG diagrams were mapped to a common reference space using a series of image processing techniques. The left-arm ICG diagram template was chosen as the reference image. Diagrams from patients with right-sided upper-limb lymphoedema were horizontally flipped to a left-arm orientation before co-registration to ensure consistent orientation. This approach was considered appropriate as prior studies have shown symmetry in normal lymphatic drainage of the upper limb when assessed according to draining lymph node fields [13]. Although more recent ICG lymphography studies have demonstrated side-to-side variation in individual lymphatic vessel pathways [14], our analysis was focused on regional drainage pathways rather than specific vessel anatomy. This study was exclusively directed at upper-limb lymphoedema, so only the upper-limb region was retained for subsequent analyses, and breast/truncal lymphoedema was excluded. Diagrams were subsequently separated into anterior and posterior views.

#### Co-registration and segmentation of ICG diagrams

Feature-based image co-registration was performed using the Oriented FAST and Rotated BRIEF (ORB) algorithm [15], which combines the FAST (Features from Accelerated Segment Test) keypoint detector with the BRIEF (Binary Robust Independent Elementary Features) descriptor. Then, the random sample consensus (RANSAC) [16] algorithm estimated the geometric transformation, aligning all annotated ICG diagrams to the template image. Following co-registration, dermal backflow regions were semi-automatically segmented using the following workflow.

First, dermal backflow regions were isolated from annotated ICG diagrams using thresholding in the CIELAB (LAB) standardized perceptual colour space defined by the International Commission on Illumination (CIE) [17]. The A-channel was used to extract the red dermal backflow regions since it represents the green-red portions of an image. Morphological refinement operations were then applied to remove small artefacts and improve continuity of segmented regions. The resulting masks were visually compared with the original diagrams and manually corrected using GNU Image Manipulation Program (GIMP) [18] software when necessary.

#### Atlas generation

After segmentation and quality checks, binary masks (0: background, 1: dermal backflow regions) were converted to numerical arrays and stacked to form a three-dimensional volume, where each slice corresponded to one ICG lymphography study. A pixel-wise mean was calculated across all masks to generate a frequency map, representing the proportion of studies exhibiting dermal backflow at each anatomical location.

Heat maps stratified by clinically defined subgroups were also generated to examine spatial variation in dermal backflow patterns across disease phenotypes. Subgroup analyses were based on MDACC stage, L-Dex ratio, LVD %, drainage regions, and presence of dermal backflow in the hand. These variables were selected as clinically relevant indicators of lymphedema severity.

### Predictive modelling of lymphedema MDACC stage

#### Feature selection

To identify the best set of features for building predictive ML models, clinical variables were correlated with MDACC stage using Spearman’s rank correlation using the R statistical package [19]. Variables were selected based on statistical significance (*p* < 0.05), strength of association, and clinical relevance, while accounting for redundancy between closely related features.

In addition to clinical variables, dermal backflow percentage was derived from each ICG diagram as a key feature for the ML models. This was calculated by adding the segmented dermal backflow areas from anterior and posterior ICG diagram views and dividing by the total area of the upper limb in the ICG diagram:

$$\:DBF\:\left(\%\right)=\:\frac{{A}_{DBF,\:ant}+\:{A}_{DBF,post}}{{A}_{arm,\:ant}+{A}_{arm,post}}\:\times\:\:100$$

where *A*_*DBF, ant*_ is the area of dermal backflow in the anterior arm, *A*_*DBF, post*_ is the area of dermal backflow in the posterior arm, *A*_*arm, ant*_ is the total area of the anterior arm, and *A*_*arm, post*_ is the total area of the posterior arm.

#### Machine learning models

Five ML models were developed to predict MDACC stage: multinomial logistic regression (MLR), random forest (RF), multi-layer perceptron (MLP), k-nearest neighbor (KNN), and support vector machine (SVM). These models were selected to represent a range of linear, non-linear, ensemble, and neural network approaches. MLR was implemented in R [20], while RF, MLP, KNN, and SVM were implemented in Python.

Model performance was evaluated using five-fold stratified cross-validation to preserve class distributions [21], with an approximate 80:20 split for training and validation in each fold. Performance was assessed using macro-averaged area under the curve (AUC), F1-score, precision, sensitivity (recall), and specificity under a one-vs-rest framework, providing balanced evaluation across stages [22]. AUC was derived from receiver operating characteristic (ROC) curves, while accuracy was calculated using the standard multi-class formulation [23]. Results are reported as mean ± standard deviation across folds.

## Results

### Patient characteristics

Patient records initially exported from the ALERT Databank included 300 ICG diagrams and 378 clinical records. Records were screened to ensure that each patient had a corresponding ICG diagram. After applying the eligibility criteria (female patients with breast cancer who developed lymphedema as a result of breast cancer treatment), 282 unique patient records and 289 ICG diagrams remained for the final analysis, including seven patients with bilateral lymphedema. The remaining dataset comprised 111 left-arm, 102 right-arm, 44 left-arm-and-torso, and 32 right-arm-and-torso diagrams. A summary of participant demographic and clinical characteristics is provided in Table 1.

**Table 1:** Demographic and clinical characteristics of the study cohort

Variable	Overall	Left	Right
Patients, *n*	282		
Affected limbs, *n*	289	148	141
Age (years), mean ± SD	58 ± 11 (range:32–82 years)		
**MDACC Staging**			
Stage 0, *n* (%)	25 (8.7)	16 (10.8)	9 (6.4)
Stage 1, *n* (%)	43 (14.9)	27 (18.2)	16 (11.3)
Stage 2, *n* (%)	120 (41.5)	59 (39.9)	61 (43.3)
Stage 3, *n* (%)	72 (24.9)	36 ( 24.3)	36 (25.5)
Stage 4, *n* (%)	29 (10.0)	17 ( 11.5)	12 (8.5)
Median (IQR): 2 (2–3)			
**LVD**			
< 0, *n* (%)	42 (14.3)		
0 ≤ LVD < 10, *n* (%)	90 (30.7)		
10 ≤ LVD < 20, *n* (%)	65 (22.2)		
≥ 20, *n* (%)	91 (31.1)		
Mean ± SD : 15.7 ± 17.4			
**L-Dex**			
< 6.5, *n* (%)	73 (24.9)		
6.5 ≤ L-Dex < 10, *n* (%)	20 (6.8)		
10 ≤ L-Dex < 20, *n* (%)	189 (64.5)		
Mean ± SD : 25.4 ± 25.5			
**Lymphatic drainage pathway (multiple nodal basins** ^**a, b**^ **)**			
Ipsilateral axilla, *n* (%)	258 (89.6)		
Clavicular, *n* (%)	88 (30.6)		
Parasternal, *n* (%)	21 (7.3)		
Parascapular, *n* (%)	14 (4.9)		
**Lymphatic drainage pathway (single nodal basin** ^**b**^ **)**			
Ipsilateral axilla, *n* (%)	170 (59.0)		
Clavicular, *n* (%)	16 (5.6)		
Parasternal, *n* (%)	2 (0.7)		
Others^c^, *n* (%)	3 (1.0)		
**Hand dermal backflow**			
None, *n* (%)	113 (39.1)		
Fingers only, *n* (%)	7 (2.4)		
Palm only, *n* (%)	58 (20.1)		
Fingers and palm, *n* (%)	111 (38.4)		

^a^ Percentages may exceed 100% as lymph may drain to more than one nodal basin. ^b^ Reported for 288 limbs; one limb had an undetermined primary drainage pathway

Note. SD = Standard Deviation; MDACC = MD Anderson Cancer Center; IQR = InterQuartile Range; LVD = Limb Volume Difference

### Heat maps of dermal backflow

#### Heat maps of dermal backflow

**Fig. 3:**
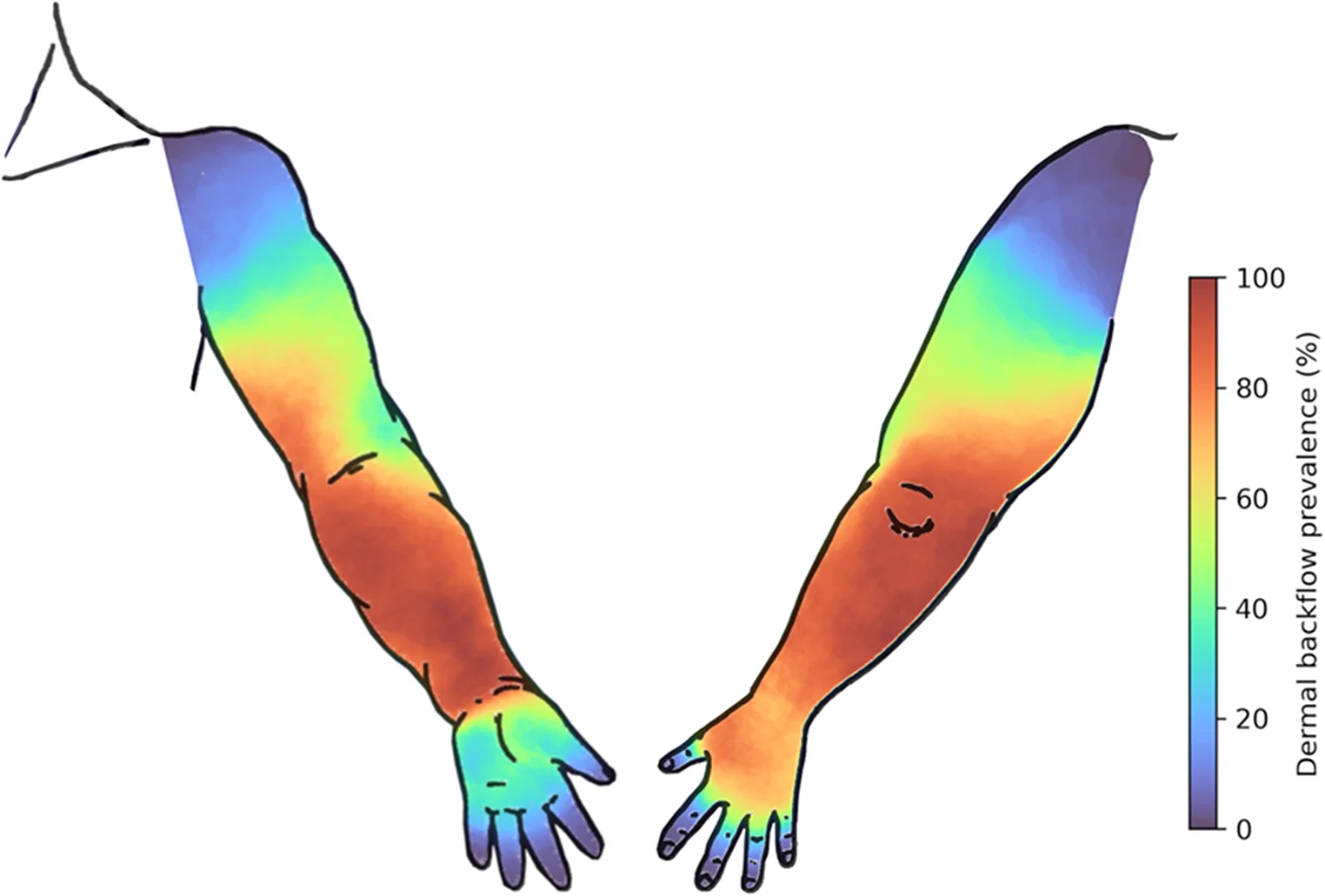
Heat maps of dermal backflow prevalence from 282 BCRL patients shown in a rainbow spectrum, anterior (**left**), posterior (**right)**

As shown in Fig. 3, a spatial heat map of dermal backflow prevalence was generated using ICG diagrams from all 282 patients with BCRL, covering 289 ICG studies in total. The heat map represents a pixel-wise normalized frequency distribution, defined as the proportion of patients exhibiting dermal backflow at each anatomical location.

Across the cohort, the spatial distribution of dermal backflow demonstrated a consistent pattern in both anterior and posterior views. In the anterior view, the highest prevalence (up to ~ 80%) was observed in the medial wrist and forearm, with prevalence decreasing proximally along the upper arm from the antecubital fossa to the axilla. In contrast, a lower prevalence (≤ 10%) was observed in the fingers and the superior shoulder region. In the posterior view, dermal backflow occurred most frequently in the olecranon region and the forearm (up to ~ 90%), with a gradual decrease in prevalence proximally along the upper arm. The axillary region and fingers exhibited the lowest frequencies (< 10%), while the thenar eminence represented the most commonly affected region of the hand.

**Fig. 4:**
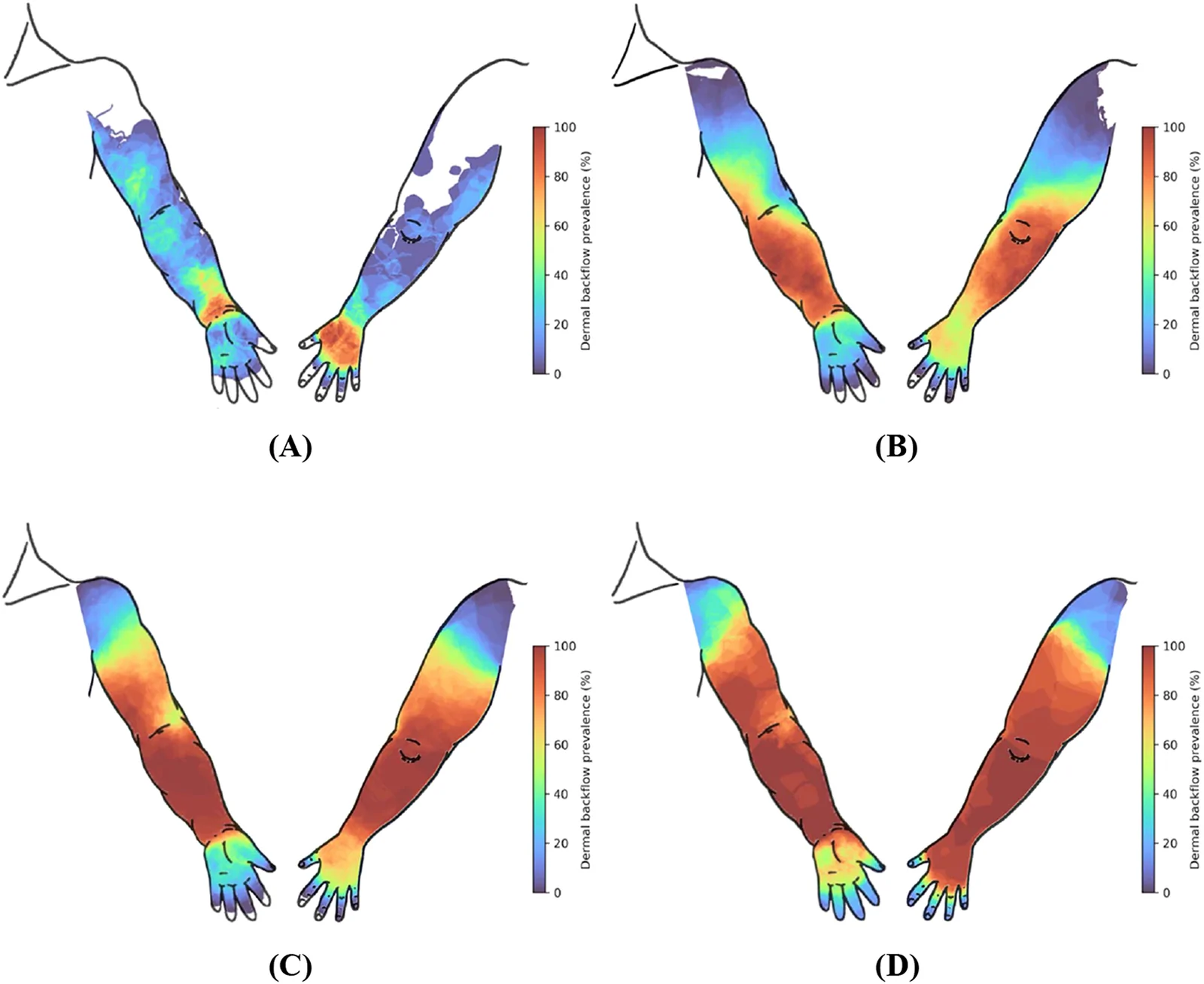
Subgroup heat maps of dermal backflow prevalence based on MDACC stage. Anterior **(left)** and posterior **(right)** views of patients with **(A)** Stage 1 (*n* = 43), **(B)** Stage 2 (*n* = 120), **(C)** Stage 3 (*n* = 72) and **(D)** Stage 4 (*n* = 29)

**Fig. 5:**
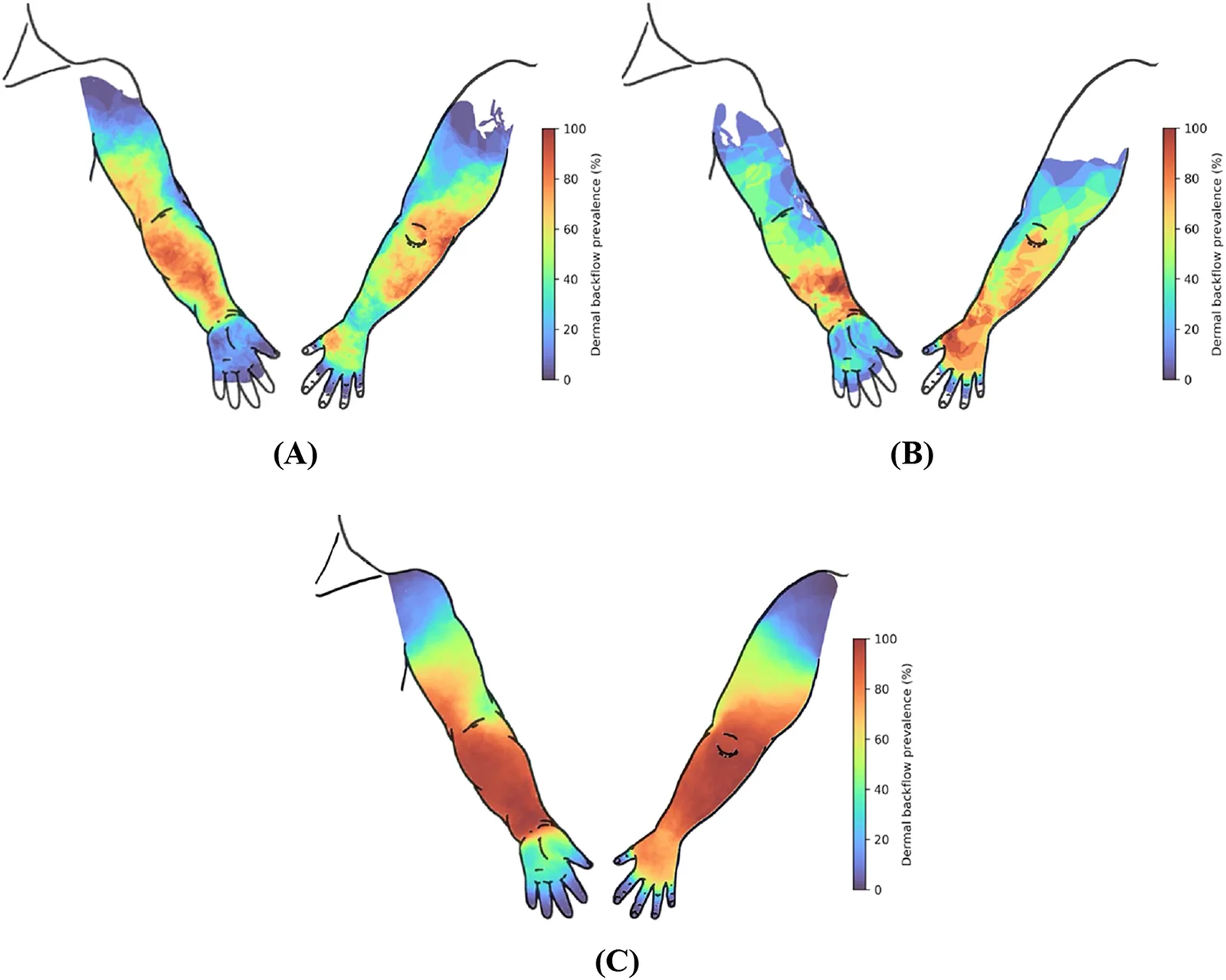
Subgroup heat maps of dermal backflow prevalence based on L-Dex ranges. Anterior **(left)** and posterior **(right)** views of patients with an L-Dex ratio of **A)** < 6.5 (*n* = 77), **B)** between 6.5 and 10 (*n* = 16), and **C)** > 10 (*n* = 187)

**Fig. 6:**
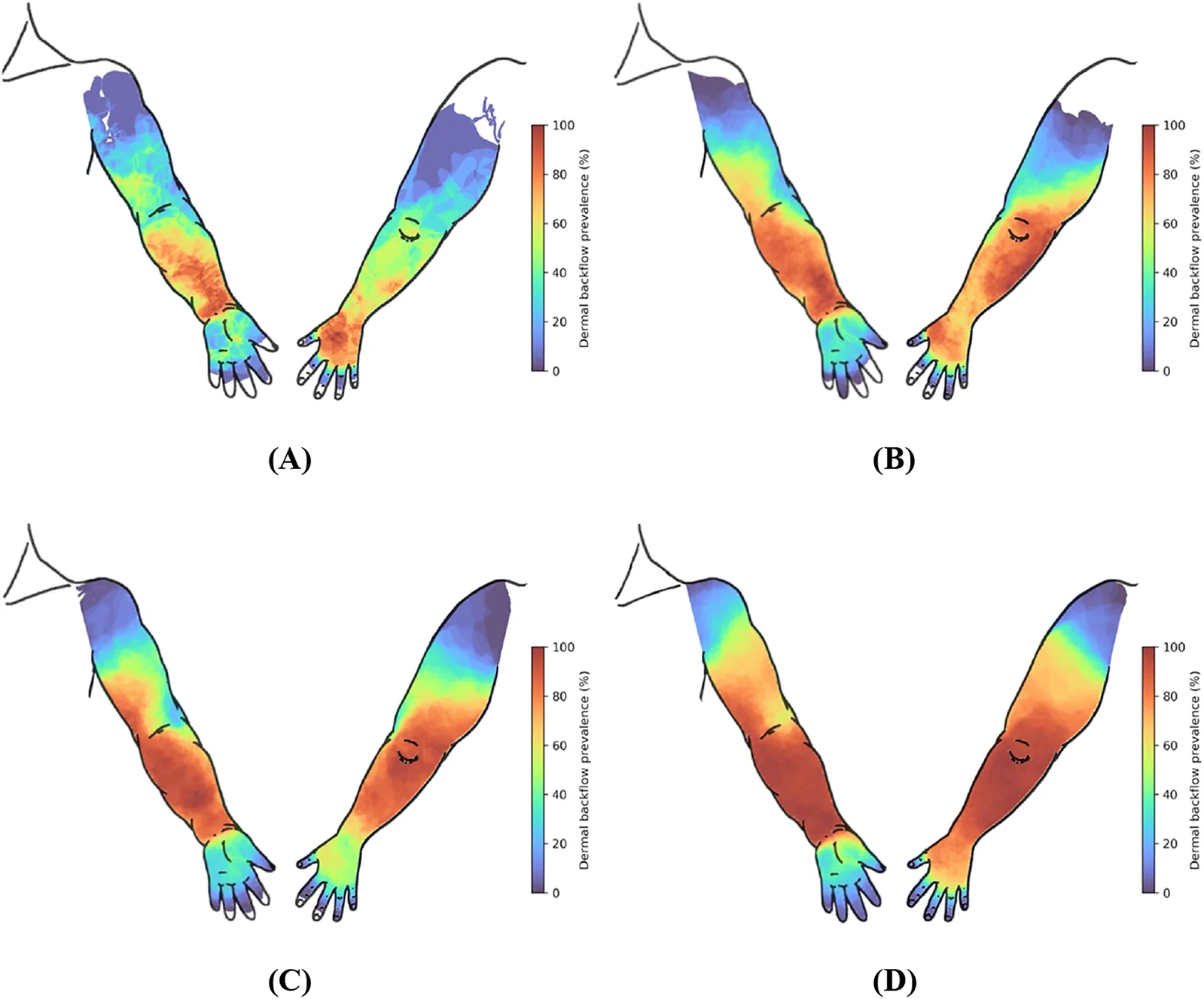
Subgroup heat maps of dermal backflow prevalence based on LVD %. Anterior **(left)** and posterior **(right)** views of BCRL patients with **(A)** LVD % < 0 (*n* = 43), **(B)** LVD % between 0 and 10 (*n* = 87), **(C)** LVD % between 10 and 20 (*n* = 61), and **(D)** LVD % > 20 (*n* = 92)

Subgroup-specific heat maps (Figs. 4, 5 and 6) revealed clear spatial differences in dermal backflow distribution across clinically defined categories. Increasing MDACC stage was associated with more extensive and proximally distributed dermal backflow patterns. Similarly, higher L-Dex ratios and greater LVD were associated with broader, more extensive regions of involvement, supporting the association with disease severity. Analysis by lymphatic drainage regions (single nodal basin) confirmed the ipsilateral axilla as the predominant pathway (Supplementary Fig. 2), while alternative drainage routes were observed in less common cases. Additionally, cases with hand involvement demonstrated more distal concentration of dermal backflow and less involvement in the upper arm (Supplementary Fig. 3). Collectively, these findings indicate that the atlas captures clinically meaningful spatial heterogeneity, reflecting both the severity and distribution of lymphatic dysfunction in BCRL.

### Classification of lymphedema stage with ML models

#### Variable selection

Table 2 presents the Spearman’s rank correlation coefficients between clinical variables and MDACC stage which were statistically significant. Dermal backflow percentage demonstrated the strongest positive correlation with stage (*ρ* = 0.90), followed by L-Dex (*ρ* = 0.67) and then LVD (*ρ* = 0.65). Moderate correlations were observed for palm and finger backflow, while variables including the number of years with lymphedema and age showed weaker associations. This process reduced the feature set from 28 to 13 variables, which were used for ML model development.

**Table 2:** Spearman’s rank correlation coefficients showing statistically significant correlations (*p* < 0.05) between MDACC stage and clinical features

Feature	Spearman ρ	*p*-value
Dermal backflow	0.90	< 0.001
L-Dex	0.67	< 0.001
LVD	0.65	< 0.001
Palm dermal backflow	0.46	< 0.001
Finger dermal backflow	0.35	< 0.001
Years with lymphedema	0.25	< 0.001
Age	0.22	< 0.001
ALND	0.18	0.003
Mastectomy	0.15	0.011
BMI	0.14	0.021
Lumpectomy	-0.14	0.019
SLND	-0.15	0.012
Year diagnosed with cancer	-0.27	< 0.001

Note. LVD = Limb Volume Difference; ALND = Axillary Lymph Node Dissection; BMI = Body Mass Index; SLND = Sentinel Lymph Node Dissection

#### Performance Evaluation

Performance of the five ML models to predict MDACC stage are shown in Table 3. All metrics achieved the standard error of less than or equal to ± 0.01%. When dermal backflow percentage was included, the RF and MLR models demonstrated the strongest overall performance. RF achieved the highest AUC (0.94) and precision (0.768), while MLR achieved the highest F1 score (0.926) and sensitivity (0.735). Both models showed comparable accuracy (0.737) and high specificity (> 0.92). MLP and SVM showed intermediate performance (AUC = 0.92), whereas KNN consistently performed the worst across all models. After excluding dermal backflow, performance decreased across all models. RF remained the best-performing model, achieving the highest AUC (0.832), accuracy (0.551), specificity (0.868), and precision (0.593). MLP achieved the highest sensitivity (0.524), while SVM showed comparable discrimination (AUC = 0.814) and balanced performance across metrics. KNN again demonstrated the lowest performance.

ROC curves were plotted to show the performance of all machine learning models (see Supplementary Fig. 4). Stage-specific ROC curves for each MDACC category demonstrated that RF had the strongest performance when dermal backflow percentage was included (see Supplementary Fig. 5), whereas RF and SVM models performed comparably when it was excluded (see Supplementary Fig. 6).

**Table 3:** Performance results from each of the ML models, with and without dermal backflow percentage included as an input variable

Dermal Backflow %	Model	AUC	Accuracy	F1	Specificity	Sensitivity	Precision
Included	MLR	0.925	0.737	**0.926**	**0.926**	**0.735**	0.741
	RF	**0.939**	**0.737**	0.712	0.924	0.704	**0.768**
	MLP	0.915	0.689	0.675	0.919	0.697	0.688
	KNN	0.834	0.564	0.510	0.874	0.508	0.567
	SVM	0.915	0.637	0.619	0.904	0.638	0.626
Excluded	MLR	0.793	0.506	**0.856**	0.856	0.464	0.518
	RF	**0.832**	**0.551**	0.494	**0.868**	0.474	**0.593**
	MLP	0.802	0.467	0.469	0.861	**0.524**	0.461
	KNN	0.725	0.474	0.390	0.849	0.409	0.419
	SVM	0.814	0.477	0.464	0.863	0.502	0.497

Note. MLR = Multinomial Logistic Regression; RF = Random Forest; MLP = Multi-Layer Perceptron; KNN = K-Nearest Neighbor; SVM = Support Vector Machine

## Discussion

This study presents a spatial atlas of dermal backflow prevalence across a cohort of 282 patients with BCRL who underwent ICG lymphography, along with subgroup heat maps based on various disease metrics. A key strength of this approach is the transformation of clinician-annotated ICG lymphography data into continuous probability maps, enabling direct visualisation of the spatial intensity and distribution of lymphatic dysfunction across the entire upper limb. Unlike conventional point-based or region-limited assessments, this framework captures spatial gradients and heterogeneity of disease expression within a unified anatomical reference.

The resulting heat maps demonstrated a clear distal-to-proximal gradient in dermal backflow intensity, with the highest probability densities observed in the anterior and posterior forearms. Additional regions of high prevalence included the wrist in the anterior view and the olecranon region and thenar eminence in the posterior view, while comparatively lower frequencies were observed proximally. These findings suggest that lymphatic dysfunction is not uniformly distributed throughout the upper limb but instead follows reproducible, region-specific spatial patterns that can be visualized and quantified using atlas-based analysis. Although distal injection sites used in ICG lymphography may enhance visualisation of dermal backflow in the hand and wrist, the consistent predominance of forearm involvement across both views suggests that the observed atlas patterns are unlikely to be explained solely by the imaging protocol.

The mechanisms underlying these regional hotspots of dermal backflow remain uncertain. However, they may reflect the organization of upper-extremity lymphatic drainage pathways and lymphosomes [24]. The antecubital region is associated with lymphatic drainage toward the epitrochlear nodes en route to axillary nodal basins. Conversely, the relatively low prevalence of dermal backflow near the axilla may reflect the normal convergence of superficial lymphatic vessels into deeper nodal structures as they approach the draining axillary lymph nodes. Further anatomical and longitudinal imaging studies are required to determine whether these hotspots represent regions of preferential vulnerability to lymphatic dysfunction.

Importantly, these spatial patterns are consistent with and extend prior physiological observations. In a study by Mayrovitz et al., they reported regional variability in superficial tissue water content in women treated for breast cancer before overt BCRL was clinically apparent [25]. The present heat map atlas builds on these findings by demonstrating that similar regional susceptibility is preserved and could be amplified in established disease. Quantitatively, distal regions such as the forearm exhibit substantially higher dermal backflow prevalence than proximal regions (approximately 70% vs. 30%), reinforcing the presence of a spatially non-uniform disease burden.

Previous work has suggested that lymphedema detection using local tissue water ratios (approximately ≥ 1.2) is typically confined to discrete anatomical measurement sites (e.g., forearm, upper arm, thorax) and implicitly assumes regional independence, potentially overlooking spatial heterogeneity [26–28]. In contrast, spatial heat maps can capture subclinical and regionally localized patterns of dermal backflow as continuous probability signals, providing a more sensitive and anatomically informative representation of disease distribution than global volume- or impedance-based metrics. As such, it may complement existing assessment techniques by providing anatomical guidance for measurement placement, particularly in consistently affected distal regions, thereby supporting improved early detection and more targeted monitoring strategies.

Subgroup analyses further demonstrated that dermal backflow extent increases with disease severity and is more prominent with elevated L-Dex and a greater LVD. A key clinical insight is that dermal backflow was often observed in limbs with minimal or negative LVD, suggesting that lymphatic dysfunction may precede measurable volumetric change. Negative LVD is likely due to arm dominance and baseline variability [29, 30]. This highlights a fundamental limitation of using conventional volume- and impedance-based assessment approaches alone, which may underestimate early or spatially localized disease. In contrast, spatial heat maps provide a more sensitive representation of disease distribution and may support earlier risk stratification and targeted monitoring.

The predictive ML modelling demonstrated that lymphedema stage could be classified with moderate to strong performance using clinical variables. The inclusion of dermal backflow percentage improved classification performance across all models. While this finding is expected, given that dermal backflow forms a key component of the MDACC staging criteria, it highlights the value of deriving quantitative measures from ICG lymphography. These results suggest that quantitative image-derived measures may provide a more objective representation of disease severity than subjective visual assessment alone, potentially improving consistency in staging and reproducibility of disease severity assessment. In clinical practice, such an approach could reduce reliance on subjective interpretation of ICG lymphography findings and streamline assessment by providing quantitative, reproducible measures of disease severity. Furthermore, the spatial heat map framework may help identify anatomical regions that are particularly susceptible to early lymphatic dysfunction, supporting earlier diagnosis and more targeted monitoring of patients at risk of progression.

Notably, ensemble and regression-based models (RF and MLR) consistently outperformed other approaches and achieved the strongest classification performance across multiple metrics, suggesting that structured and interpretable models remain highly effective for clinically constrained datasets. This is broadly consistent with previous studies [31–33] reporting strong performance of logistic regression–based approaches. However, prior work has largely focused on symptom-based prediction or BCRL occurrence rather than imaging-derived staging [32, 34, 35]. In contrast, the present study extends this evidence by applying these models to quantitative imaging features, including dermal backflow, for MDACC stage classification. The superior performance of RF and MLR, particularly when combined with the dermal backflow percentage, supports their suitability for translational applications in which interpretability is essential. In contrast, non-linear models such as MLP and SVM demonstrated higher sensitivity for advanced (MDACC Stage 4) lymphoedema. However, given that advanced disease is typically clinically apparent, this advantage is unlikely to outweigh their lower overall classification performance compared with MLR and RF.

While some studies have reported comparable performance of more complex models such as MLPs and artificial neural networks (ANNs) with MLR [32, 34], their reduced interpretability may limit clinical applicability compared with regression- and tree-based methods. In addition, methodological differences across studies, particularly in input feature sets such as the inclusion of blood biomarkers in some RF-based models, likely contribute to variations in reported performance and limit direct comparability. Together, these findings highlight the importance of model interpretability and consistent feature sets when evaluating ML approaches for clinically meaningful staging of BCRL.

Several limitations should be acknowledged. The atlas was derived from clinician-annotated diagrams rather than raw ICG lymphography images and therefore reflects clinical interpretation of dermal backflow rather than the original imaging data. The diagrams were produced by four clinicians at the ALERT Centre, three of whom were trained by HS using a consistent imaging and annotation approach. Although this standardised training likely reduced inter-clinician variability, differences in image interpretation and annotation may still have influenced the spatial representation of dermal backflow. Seven out of 282 patients (2.5%) contributed bilateral limbs, introducing the potential for non-independence between observations, though this is unlikely to have materially affected the results. Furthermore, achieving a fully automated registration and segmentation pipeline would streamline and save time in future work. As an exploratory step towards reducing manual intervention, a U-Net–based deep learning model was initially evaluated because of its established performance in medical image segmentation [36]. Although the U-Net model achieved excellent cross-validation performance, performance decreased when applied to the full dataset, particularly in distal hand regions, and therefore was not used. Further improvements may be achieved through larger annotated datasets and more diverse training examples.

In addition, future work should focus on analysing raw or volumetric imaging data within a unified three-dimensional framework. Such approaches would also improve the calculation of dermal backflow percentage, which was derived from two-dimensional ICG diagrams and therefore provided only an approximation of the true three-dimensional skin surface. As with any projection-based analysis, this approach did not account for surface curvature or perspective-related foreshortening, which may lead to minor inaccuracies in the quantification of dermal backflow distribution. The development of fully automated pipelines using larger, multi-centre datasets and advanced architectures capable of capturing small, highly variable regions would further improve robustness and generalizability of the proposed framework. Future extensions of this work could also examine whether different breast cancer treatment, especially sentinel lymph node dissection and axillary lymph node dissection, are associated with differences in dermal backflow prevalence in patients.

## Conclusion

This study demonstrates that quantitative analysis of ICG lymphography in a cohort of patients can improve the characterisation and staging of BCRL. The imaging-based atlas identified consistent spatial patterns of dermal backflow in the upper limb, and ML-based models to classify lymphedema stage improved when dermal backflow metrics were included. These findings highlight the potential of spatial imaging biomarkers to support more objective and standardized lymphedema assessment. The atlas and quantitative analysis framework have several potential applications. The dermal backflow atlas could provide clinicians with a reference for understanding common patterns of lymphatic dysfunction and facilitate comparison with individual patient ICG lymphography findings. Quantitative measures of dermal backflow may also support more objective and reproducible assessment of disease severity, complementing existing clinical evaluation rather than replacing it. Together, these tools provide a foundation for more standardised assessment of BCRL and may support future decision-support systems and more personalized surveillance strategies as larger datasets and prospective validation become available. Future work should focus on external validation and clinical translation in larger, multi-centre cohorts.

## Supplementary Information

Below is the link to the electronic supplementary material.

Supplementary Material 1

## Data Availability

The data that support the findings of this study were obtained from the ALERT Databank. Restrictions apply to the availability of these data including personal health information, which were accessed under permission for the current study and are therefore not publicly available. Access to the ALERT Databank is subject to the databank’s governance and approval processes. Data may be available from the ALERT Databank upon reasonable request and with appropriate authorization.
