# Supplementary material for: A dermal backflow atlas of lymphatic dysfunction in breast cancer-related lymphedema with machine learning-based stage prediction
Source: Breast Cancer Res Treat. 2026 Oct 7;219(3):28. doi: 10.1007/s10549-026-08093-2 (PMC13645920; doi:10.1007/s10549-026-08093-2)
Supplement: Supplementary file 1 [file 10549_2026_8093_MOESM1_ESM.docx]

**Supplementary Materials**

**A Dermal Backflow Atlas of Lymphatic Dysfunction in Breast Cancer-Related Lymphoedema with Machine Learning-Based Stage Prediction**

Lalida Sutejitsiri^1^ **⋅** Hiroo Suami^2^ **⋅** Poul Nielsen^1,3^ **⋅** Anthony Phillips^4^ **⋅** Hayley Reynolds^1^

^1^ Auckland Bioengineering Institute, The University of Auckland, Auckland, New Zealand

^2^ Australian Lymphedema Education Research and Treatment Program (ALERT), Department of Health Sciences, Faculty of Medicine, Health and Human Sciences, Macquarie University, Sydney, Australia

^3^ Engineering Science and Biomedical Engineering, Faculty of Engineering, The University of Auckland, Auckland, New Zealand

^4^ School of Biological Sciences, Faculty of Science, The University of Auckland, Auckland, New Zealand

**Supplementary Figure 1** Staging and severity scale of lymphoedema from patterns found in ICG lymphography according to MDACC based on the number of patent lymph vessels, amount of dermal backflow, and contractility of lymphatic vessels (Adapted from Nguyen et al. [19] with permission from John Wiley and Sons).

**A)**

**B)**

**Supplementary Figure 2** Subgroup heat maps of dermal backflow prevalence for patients with a single drainage region. **A)** ipsilateral axilla only (n = 170) and **B)** the clavicular node field only (n = 16).

**A)**

**B)**

**C)**

**D)**

**Supplementary Figure 3** Subgroup heat maps of dermal backflow prevalence displaying patients with **A)** no hand backflow (n = 113), **B)** backflow into the fingers (n = 7), **C)** backflow into the palms (n = 58), and **D)** backflow into both fingers and palms (n = 111).

**Supplementary Figure 4** ROC curves and AUCs of five ML models averaged across all MDACC stages, showing **Top)** results with dermal backflow (%) included and **Bottom)** results without dermal backflow (%).

**D)**

**C)**

**A)**

**B)**

**E)**

**Supplementary Figure 5** ROC curves and AUCs of five ML models including DBF % across all MDACC stages, showing **A)** stage 0, **B)** stage 1, **C)** stage 2**, D)** stage 3, and **E)** stage 4.

**C)**

**D)**

**B)**

**A)**

**E)**

**E)**

**Supplementary Figure 6** ROC curves and AUCs of five ML models excluding DBF % across all MDACC stages, showing **A)** stage 0, **B)** stage 1, **C)** stage 2**, D)** stage 3, and **E)** stage 4.
